# *Arabidopsis* IQM4, a Novel Calmodulin-Binding Protein, Is Involved With Seed Dormancy and Germination in *Arabidopsis*

**DOI:** 10.3389/fpls.2018.00721

**Published:** 2018-06-05

**Authors:** Yu Ping Zhou, Jing Hui Wu, Wen Hui Xiao, Wei Chen, Qiong Hua Chen, Tian Fan, Chu Ping Xie, Chang-En Tian

**Affiliations:** ^1^Guangzhou Key Laboratory for Functional Study on Plant Stress-Resistant Genes, Guangzhou University, Guangzhou, China; ^2^School of life Sciences, Guangzhou University, Guangzhou, China

**Keywords:** abscisic acid, calmodulin-binding protein, germination, *IQM4*, seed dormancy

## Abstract

Seed dormancy and germination are regulated by complex mechanisms controlled by diverse hormones and environmental cues. Abscisic acid (ABA) promotes seed dormancy and inhibits seed germination and post-germination growth. Calmodulin (CaM) signals are involved with the inhibition of ABA during seed germination and seedling growth. In this study, we showed that *Arabidopsis thaliana* IQM4 could bind with calmodulin 5 (CaM5) both *in vitro* and *in vivo*, and that the interaction was the Ca^2+^-independent type. The IQM4 protein was localized in the chloroplast and the *IQM4* gene was expressed in most tissues, especially the embryo and germinated seedlings. The T-DNA insertion mutants of *IQM4* exhibited the reduced primary seed dormancy and lower ABA levels compared with wild type seeds. Moreover, *IQM4* plays key roles in modulating the responses to ABA, salt, and osmotic stress during seed germination and post-germination growth. T-DNA insertion mutants exhibited ABA-insensitive and salt-hypersensitive phenotypes during seed germination and post-germination growth, whereas *IQM4*-overexpressing lines had ABA- and osmotic-hypersensitive, and salt-insensitive phenotypes. Gene expression analyses showed that mutation of *IQM4* inhibited the expression of ABA biosynthetic genes *NCED6* and *NCED9*, and seed maturation regulators *LEC1, LEC2, ABI3*, and *ABI5* during the silique development, as well as promoted the expression of *WRKY40* and inhibited that of *ABI5* in ABA-regulated seed germination. These observations suggest that IQM4 is a novel Ca^2+^-independent CaM-binding protein, which is positively involved with seed dormancy and germination in *Arabidopsis*.

## Introduction

Seed dormancy and germination are distinct physiological processes, which are partly controlled by organized alterations in the biosynthetic and signaling pathways for major plant hormones including abscisic acid (ABA) and gibberellins (GAs) (Finch-Savage and Leubner-Metzger, 2006; Finkelstein et al., 2008; Shu et al., 2016). In *Arabidopsis thaliana* (*Arabidopsis*), seed maturation and the induction of dormancy are genetically controlled by a network of transcription factors, including the *ABSCISIC ACID INSENSITIVE 3*(*ABI3*), *FUSCA3* (*FUS3*), and *LEAFY COTYLEDON 2*(*LEC2*) clade of B3 domain transcription factors, and two LEAFY COTYLEDON 1 (LEC1)-type HAP3 family CCAAT-binding factors, *LEC1* and *LEC1-LIKE* (*L1L*), which are designated as the LAFL network (Raz et al., 2001; Jia et al., 2013). The LAFL network participates in the integration of hormonal and intrinsic developmental signals during seed development (Holdsworth et al., 2008; Jia et al., 2014). All four *abi3, fus3, lec2*, and *lec1* mutants are severely affected during seed maturation and they share some common phenotypes, such as the reduced seed dormancy (Raz et al., 2001) and the decreased expression of seed storage proteins (Gutierrez et al., 2007).

When the developing embryo enters the maturation phase, the high ABA/GA ratio promotes seed maturation and induces dormancy (Finkelstein et al., 2008; Holdsworth et al., 2008). The endogenous ABA level is regulated via the dynamic balancing of biosynthesis and catabolism, including feedback induction of catabolism (Nambara and Marion-Poll, 2005; Nonogaki et al., 2014). Nine-*cis*-epoxycarotenoid dioxygenase (NCED) catalyzes the rate-limiting step in ABA biosynthesis (Martinez-Andujar et al., 2011), among the five members (*NCED2, 3, 5, 6*, and *9*) of the *Arabidopsis NCED* family, *AtNCED5, AtNCED6*, and *AtNCED9* primarily regulate seed development and dormancy (Tan et al., 2003; Lefebvre et al., 2006; Frey et al., 2012). In contrast, ABA-8′-hydroxylase (encoded by *CYP707A1-4*) is considered to be the key enzyme in ABA catabolism (Kushiro et al., 2004). Each CYP707A plays a different role in the control of seed dormancy and germination in *Arabidopsis*. CYP707A1 and CYP707A2 are the major isoforms during mid-maturation and late maturation, respectively (Okamoto et al., 2006).

In addition to ABA levels, ABA signaling plays a pivotal role in seed maturation and germination. Genetic analyses have identified several major classes of ABA signal regulators, such as protein phosphatases and kinases, transcription factors, and RNA processing enzymes (Finkelstein et al., 2008). ABA acts through the pyrabactin resistance 1/PYR1-like/regulatory components of ABA receptor (PYR/PYL/RCAR)–protein phosphatase 2C(PP2C)–SNF1-related kinase 2 (SnRK2) signaling cascade (Cutler et al., 2010; Hubbard et al., 2010). The PP2C proteins, ABI1 and ABI2, bind to ABA receptors to inhibit ABA signaling. The SnRK2 members act as positive regulators in the ABA signaling pathway. Moreover, several key transcription factors located downstream of the ABA signaling pathway play crucial roles in seed dormancy and germination. *Arabidopsis* ABSCISIC ACID INSENSITIVE 4 (*ABI4*) and ABSCISIC ACID INSENSITIVE 5 (*ABI5*) encode different transcription factors for the AP2 domain and bZIP domain families, respectively (Finkelstein et al., 2002; Nambara and Marion-Poll, 2003). ABI4 positively regulates seed dormancy and ABA signaling by binding directly to the promoters of *CYP707A1* and *CYP707A2* (Shu et al., 2013). ABI5 is involved with seed maturation and ABA signaling in vegetative tissue (Finkelstein and Lynch, 2000). *ABI5* expression is regulated by *ABI3* and ABA, and ABI5 controls the expression of late embryogenesis abundant (LEA) protein genes in the seed by binding directly to the ABA-responsive element (ABRE) *cis*-element, which is present in the promoters of several *LEA* genes such as *Em1* and *Em6* (Bensmihen et al., 2002; Finkelstein et al., 2005). Some WRKY transcription factors have been identified as important components of the ABA signaling pathway (Rushton et al., 2012). Loss-of-function mutants of *WRKY2, WRKY40*, and *WRKY63* all yield the ABA-hypersensitive phenotype during seed germination and post-germination growth in *Arabidopsis*. WRKY40 directly and negatively regulates the expression of *ABI4* and *ABI5*, whereas *WRKY63* acts downstream of *ABI5* (Jiang and Yu, 2009; Chen et al., 2010; Shang et al., 2010). In addition, *WRKY41* directly regulates the expression of *ABI3* in maturing and imbibed seeds, thereby controlling both seed dormancy and thermoinhibition (Ding et al., 2014).

Ca^2+^ is a universal second messenger that acts as a mediator of stimulus–response couplings during the regulation of diverse cellular functions (Trewavas and Malho, 1998; Rudd and Franklin-Tong, 2001; McAinsh and Pittman, 2009). The intracellular Ca^2+^ concentration is often elevated significantly in response to environmental stimuli and intrinsic developmental cues. Ca^2+^ signals are sensed and translated into appropriate cellular responses by various Ca^2+^ binding proteins and their downstream targets. Calmodulin (CaM) and CaM-Like (CML) are the major Ca^2+^ binding proteins. CaM/CML physically associates with numerous targets including protein kinases and phosphatases, metabolic enzymes, transcription factors, heat shock proteins, transporters, channels, and a variety of proteins with unknown functions (Bouché et al., 2005; Bender and Snedden, 2013; Zeng et al., 2015). In *Arabidopsis*, seven *CaM* genes encode four CaM isoforms (CaM1/4, CaM2/3/5, CaM6, and CaM7) and 50 genes encode CML proteins, which contain variable numbers of EF hands; all share at least 16% of their overall sequence identity with canonical CaM (McCormack and Braam, 2003; McCormack et al., 2005). CaMs/CMLs interact with downstream target proteins via the CaM-binding domain (CaMBD), which comprises a stretch of 16–35 amino acids. The CaMBD usually belongs to one of three groups: 1-5-10, 1-8-14, and IQ motifs. The first two are Ca^2+^ dependent; the IQ motif contains an IQxxxRGxxxR consensus sequence where “I” can be replaced with “FLV” and “x” represents any amino acid residue, where it is known to appear in tandem repeats and bind multiple CaM molecules in a predominantly Ca^2+^-independent manner (Bähler and Rhoads, 2002; Hoeflich and Ikura, 2002).

Several studies have indicated that CaM/CML signals are involved in ABA-induced inhibition of seed germination and seedling growth. For instance, the transcription regulators, *AtCAM7* and *HY5*, work together to control light-induced seedling development (Kushwaha et al., 2008). Moreover, *cam7* mutants are more susceptible to ABA-inhibited seed germination, and CAM7 and HY5 work in an antagonistic manner (Abbas and Chattopadhyay, 2014; Abbas et al., 2014). *AtCML9* is readily induced by abiotic stress and ABA, and the *cml9* null mutant exhibits a hypersensitive response to ABA during seed germination and seedling growth (Magnan et al., 2008). *AtCML24-*underexpressing transgenic lines are resistant to ABA-induced inhibition of seed germination and seedling growth (Delk et al., 2005). *AtCML37* and *AtCML42* are involved with the drought stress response but have antagonistic effects, where CML37 promotes ABA accumulation under drought stress but CML42 inhibits this accumulation (Vadassery et al., 2012; Scholz et al., 2015). A novel *CML* gene, OsMSR2, was isolated from *Oryza sativa* and transgenic lines of this gene exhibited hypersensitivity to ABA during seed germination and post-germination growth in *Arabidopsis* (Xu et al., 2011). In CaM/CML signaling, only a few CaM binding proteins (CaMBPs) have been shown to function during ABA signaling. For example, AtCBP60g, a CaM-binding transcription factor, positively regulates the drought stress response, and transgenic plants that overexpress CBP60g exhibit hypersensitivity to ABA and enhanced drought tolerance (Wan et al., 2012). However, the roles of CaMBPs in ABA-regulated seed dormancy and germination still remain unclear. Therefore, it is important to identify the downstream targets of the CaM/CMLs mentioned above during ABA-regulated seed germination, and to elucidate the other components of CaM signaling that regulate seed dormancy and germination.

Previously, we identified a novel IQ-motif-containing CaMBP family, IQM, in *Arabidopsis*, where the members share sequence homology with pea heavy metal-induced protein 6 and a ribosome-inactivating protein, trichosanthin. The diverse expression patterns of each member of the IQM family suggest that each *IQM* gene may play a distinct role in plant development and the response to environmental cues (Zhou et al., 2010). Furthermore, IQM1 was found to be a Ca^2+^-independent CaMBP that acts as a key player in the modulation of stomata movement (Zhou et al., 2012). IQM5 was found to be involved with the regulation of flowering (Gong et al., 2017). IQM4 shares the highest identity (80%) with IQM1 in the IQM family. The expression of *IQM4* is affected by light, mannitol, salt, and ABA (Zhou et al., 2010), but its function remains unknown. In this study, we showed that IQM4 has a CaM-binding activity; we also detected the subcellular localization of IQM4 protein and elucidated that it is involved with seed dormancy and germination, possibly by the modulation of ABA biosynthesis and ABA signaling in the seed.

## Materials and Methods

### Plant Materials and Growth Conditions

The *Arabidopsis thaliana* ecotype Columbia (Col-0) was used in this study. Seeds of *iqm4-1* (SALK_101916) and *iqm4-2* (SALK_120435) were obtained from the Arabidopsis Biological Resource Center (ABRC, Ohio State University, Columbus, OH, United States^1^). Homozygotes of the mutant individuals were validated by PCR using the IQM4-specific primer pair F1/R1 and the T-DNA left border primer LBb1 (primer sequences are listed in **Supplementary Table S1**) and the exact position was determined by sequencing. For mature plants, seeds were imbibed in the dark at 4°C for 3 days and then grown in soil in a culture room at 22 ± 1°C and 70% relative humidity (RH) under a 16 h light/8 h dark photoperiod with a photon fluency rate of 100 μmol m^-2^s^-1^. The pot-grown plants were watered every 2 or 3 days. The seeds were sometimes germinated and grown aseptically on solid medium containing half-strength Murashige-Skoog (MS) salts (Murashige and Skoog, 1962), 1% (w/v) sucrose, and agar (pH 5.8) in the culture room after surface sterilization and stratification in darkness for 3 days at 4°C.

### Yeast Two-Hybrid Assays and Site-Directed Mutation of *IQM4*

The Matchmaker Gal4 two-hybrid system (Clontech, Palo Alto, CA, United States) was used according to the manufacturer’s instructions. The full length open reading frame (ORF) of *CaM5* (At2g27030) was generated by RT-PCR using CaM5-F/R primers (**Supplementary Table S1**), and fused into the *EcoR* I and *Sal* I sites in the bait plasmid pGBKT7. The full length ORF of *IQM4* (At2g26190) was amplified by RT-PCR using IQM4-F3/R3 primers (**Supplementary Table S1**) and cloned into the *Nde* I and *Xma* I sites in the prey plasmid pGADT7. The constructed vector pGADT7-IQM4 was used as the template, and primer pairs were designed based on the deleted or substituted DNA sequences encoding the IQ motif in IQM4 (del143–144 and del143–144 antisense for *IQM4*^Δ143-144^; L143N and L143N antisense for *IQM4*^L143N^) (**Supplementary Table S1**), where the mutagenesis program was employed according to the manufacturer’s instructions for the QuikChange Lightning Site-Directed Mutagenesis Kit (Agilent Technologies, Palo Alto, CA, United States). The two types of *IQM4* mutants were designated as *IQM4*^Δ143-144^ and *IQM4*^L143N^. Yeast transformants were selected on synthetic dropout medium (SD medium) according to the manufacturer’s instructions. Different combinations of plasmids were transformed into the yeast strain AH109. Transformants were plated onto Leu-Trp-deficient (SD-2) and Leu-Trp-His-Ade-deficient (SD-4) media, and grown for 5–7 days at 30°C. The empty vectors pGBKT7 and pGADT7 were used a negative controls, and pGBKT-53 and pGADT7-T were used as positive controls. The DNA sequences of all the DNA constructs described in this study were verified by DNA sequencing.

### CaM Gel Overlay Assay

This assay was performed according to a previously reported procedure (Zhou et al., 2012). The partial ORF of *IQM4* (encoding amino acid residues 1–160; *IQM4*ΔC) was generated by RT-PCR using *IQM4*-F4/R4 primers (**Supplementary Table S1**) and cloned into the *Nco* I and *Hind* III sites of pET32a(+) (Novagen-Merck, Darmstadt, Germany). pET32a-IQM4ΔC was transformed into the *E. coli* strain BL21 (DE3) pLys S. Protein expression was induced with 1 mM isopropyl-1-thio-β-D-galactopyranoside for 4–5 h at 30°C. The crude proteins were electrophoresed by 15% sodium dodecyl sulfate-polyacrylamide gel electrophoresis (SDS-PAGE) and detected by western blotting using 6× His antibody (**Supplementary Figure S2**). Fifty micrograms of the crude separated protein from induced bacterial cells harboring the pET32a plasmid (CK) and pET32a-IQM4ΔC plasmid were transferred to nitrocellulose membranes and were probed with the biotinylated CaM5 as described by Zhou et al. (2012) in the presence of 1 mM CaCl_2_ or 5 mM ethylene glycol tetraacetic acid (EGTA).

### Bimolecular Fluorescence Complementation (BiFC) Assay

The full-length ORFs of *IQM4* and *CaM5* were generated by RT-PCR using IQM4-F5/R5 primers and CaM5-F/R primers, respectively (**Supplementary Table S1**), for the BiFC assay using onion epidermal cells. *IQM4* ORF was fused into the *Hind* III and *Xma* I sites downstream of the *nEYFP* gene in the pSATN-nEYFP-C1 vector, and *CaM5*ORF was inserted into the *EcoR* I and *Sal* I sites downstream of *cEYFP* in the pSATN-cEYFP-C1 vector (Citovsky et al., 2006). The plasmid combinations were introduced into onion epidermal cells with the Model PDS-1000/He Biolistic Particle Delivery System (Bio-Rad) using 1-μm diameter gold particles, as described in the manufacturer’s instructions. After bombardment, the onion epidermal tissues were incubated overnight at 25°C on solid Murashige-Skoog (MS) medium in the dark, and observed with an epifluorescence microscope (TE2000-U, Nikon, Tokyo, Japan).The plasmid combinations were introduced into *Arabidopsis* mesophyll protoplasts as described by Sheen (2001). After incubating for 18 h at 23°C in the dark, the protoplasts were examined for YFP signal using confocal laser scanning microscopy (LSM 7 DUO, Zeiss).

### Subcellular Localization of IQM4

The 1874-bp putative promoter was amplified by PCR with the pIQM4-F/R primers (**Supplementary Table S1**) from genomic DNA, and cloned into the *BamH* I and *Xma* I sites upstream of the *green fluorescent protein* (*GFP*) gene in the modified pEGFP vector (Clontech) to generate the control vector pIQM4:GFP. The full-length ORF of *IQM4* with the stop codon deleted was amplified by RT-PCR using IQM4-F6/R6 primers (**Supplementary Table S1**), and inserted into the *Xma* I and *Nco* I sites upstream of the *GFP* gene of the control vector pIQM4:GFP to generate the fusion gene vector pIQM4:IQM4-GFP. Both the fusion gene vector pIQM4:IQM4-GFP and the control vector pIQM4:GFP plasmids were introduced into *Arabidopsis* mesophyll protoplasts as described by Sheen (2001). After incubating for 60 h at 23°C in the dark, the protoplasts were examined for GFP signal using confocal laser scanning microscopy (LSM 7 DUO, Zeiss).

### Histochemical β-Glucuronidase (GUS) Staining

To generate the pBI101-pIQM4:GUS vector, the 1874-bp putative promoter was amplified by PCR with pIQM4-F/R primers (**Supplementary Table S1**) from genomic DNA, and then cloned into the *Xba* I and *Xma* I sites upstream of the *GUS* gene in the binary vector pBI101 (Clontech). Subsequently, pBI101-pIQM4:GUS was transferred into *Agrobacterium tumefaciens* strain GV3101 and then introduced into *Arabidopsis* wild type Col using the floral dip method (Clough and Bent, 1998). Three generations of transgenic seeds were selected on half-strength MS medium (1/2 MS) containing 50 mg/L kanamycin, and the T3 homozygous line containing a single insertion was used for further detailed analysis. Histochemical GUS staining was performed as described by Jefferson et al. (1987). Seedlings or tissues were incubated for 12–16 h at 37°C in reaction buffer containing 2 mM 5-bromo-4-chloro-3-indolyl-b-D-glucuronic acid. The plant pigments were removed with ethanol and GUS staining was recorded under a dissecting microscope (SMZ800, Nikon).

### Quantification of ABA and GA3

Five-hundred milligram of powdered seeds ground in liquid nitrogen were homogenized in 5 mL of extraction buffer containing isopropyl alcohol/hydrochloric acid, before shaking for 30 min at 4°C and adding 10 mL of dichloromethane, shaking for 30 min at 4°C, and centrifugation for 5 min at 14000 ×*g* and 4°C. The lower organic phase was sucked out and blown dry with nitrogen, before dissolving with 400 μL of methanol (0.1% formic acid). The solution was then filtered through 0.22-μm membranes. The elute was injected into a liquid chromatography-tandem mass spectrometry system comprising a high performance liquid chromatograph (Agilent 1290 Infinity II) and a mass spectrometer (AB SCIEX QTRAP^®^ 6500). Four biological replicates were performed.

### Seed Germination and Primary Root Length Assays

For the seed germination assays, approximately 100 wild type and *iqm4* mutants seeds were sown on 1/2 MS medium containing 1% sucrose with different concentrations of ABA (Sigma-Aldrich) (from 0 to 4 μM), NaCl (from 0 to 150 mM), or mannitol (from 0 to 400 mM). The seeds were incubated for 3 days in the dark at 4°C to disrupt any residual dormancy before being transferred to growth chambers. Germination was scored daily. A seed was considered to have germinated when the radicle protruded through the seed coat. The seedling with green cotyledons was used for measuring the rate of cotyledon greening. In assays of the primary root length, 5-day-old seedlings grown vertically in 1/2 MS plates were transferred onto new plates supplemented with ABA (0, 3, or 6 μM). Each square plate contained 12 seedlings. The primary root length was measured using Scion Image software after 7 days. All experiments were repeated with at least four different batches of seeds. Photographs were taken using the Nikon Camera and the results from one representative experiment are shown. The germination rate, primary root length, and standard errors (SEs) were calculated based on the results of four independent experiments. The numerical data were subjected to statistical analyses using EXCEL and KaleidaGraph statistical software.

### Generation of *IQM4*-Overexpressing Transgenic Plants

For generating the pBI121-p35S:*IQM4* construct, the full length ORF of *IQM4* was amplified by RT-PCR usingIQM4-F7/R7 primers (**Supplementary Table S1**) and cloned into the *Xba* I and *Xma* I site in the binary vector pBI121 (Clontech). The construct pBI121-p35S:IQM4 was transferred into *Agrobacterium tumefaciens* strain GV3101 and then introduced into Arabidopsis wild type Col-0 using the floral dip method (Clough and Bent, 1998). Three generation of transgenic seeds were selected on 1/2 MS medium containing 50 mg/L kanamycin. Transcripts of *IQM4* in T3 homozygous lines containing a single insertion were assessed using RT-PCR and real time RT-PCR (qRT-PCR) and used for more detailed phenotypic analysis.

### Gene Expression Analysis

Total RNA was isolated from siliques and seedlings using the TransZol Plant reagent (TransGen, China). First-strand cDNA synthesis was performed according to the instructions provided with the PrimeScript^TM^ RT reagent Kit with gDNA Eraser (Code RR047Q, Takara, China). Total RNA (2 μg) was digested using gDNA Eraser to remove genomic DNA and subjected to reverse transcription using PrimeScript RT Enzyme. The resulting cDNA was used as template in both RT-PCR and real-time RT-PCR. Fifty nanograms of cDNA was subjected to PCR with Ex Taq HS (Takara, China) using the IQM4-F1/R1 primers given in **Supplementary Table S1**. The expression levels of *β-ATPase* (At5g08670) were monitored as an internal control. For real-time RT-PCR, 20 ng of cDNA was used in each reaction system and all of the reactions were performed in quadruplicate using TB Green^TM^ Premix Ex Taq^TM^II (Tli RNaseH Plus) (Takara, China) and Applied Biosystems 7300 Fast Real-Time PCR System (Life Technologies). Gene expression levels were quantified in the logarithmic phase using the expression of the *ACTIN2* housekeeping gene as an internal control. The genes quantified were *ACTIN2* (AT3G18780), *IQM4* (AT2G26190), *NCED3* (AT3G14440), *NCED6* (AT3G24220), *NCED9* (AT1G78390), *CYP707A1* (AT4G19230), *CYP707A2* (AT2G29090), *CYP707A3* (AT5G45340), *LEC1* (AT1G21970), *LEC2* (AT1G28300), *FUS3* (AT3G26790), *ABI3* (AT3G24650), *ABI4* (AT2G40220), *ABI5* (AT2G36270), *WRKY40* (AT1G80840), *ABI1* (AT4G26080), *ABI2* (AT5G57050), *HAB1* (AT1G72770), *HAB2* (AT1G17550), *SnRK2.2* (AT3G50500), *SnRK2.3* (AT5G66880), and *RAB18* (AT5G66400). All of the primer sequences are given in **Supplementary Table S2**.

## Results

### *IQM4* Encodes a Novel Ca^2+^-Independent CaMBP

**FIGURE 1:**
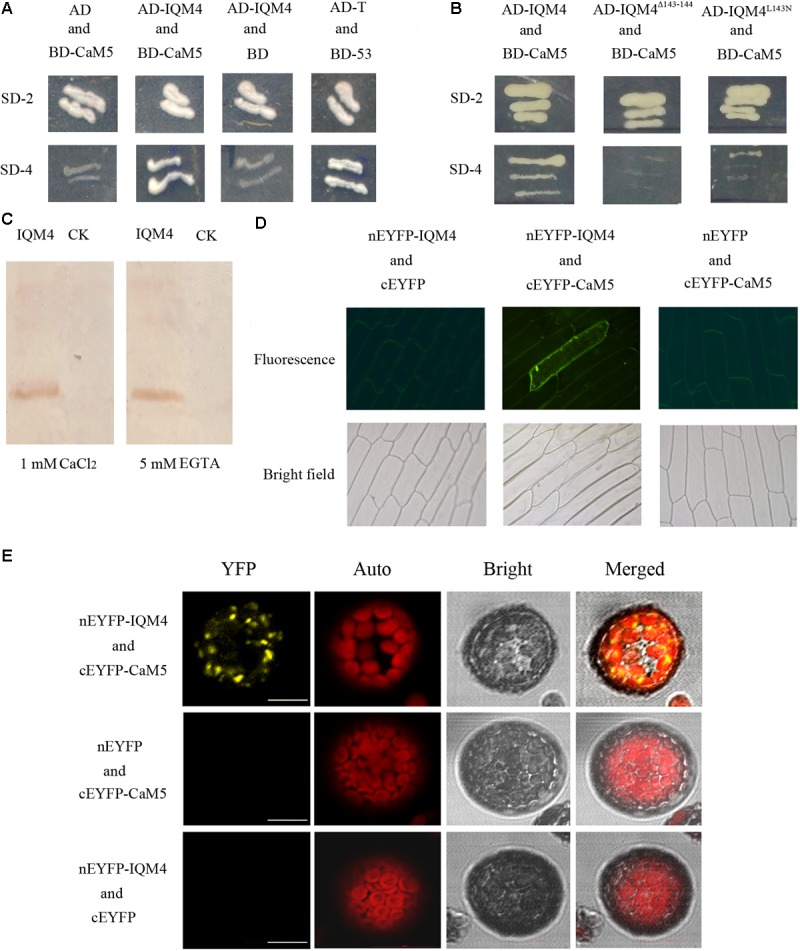
IQM4 is a Ca^2+^-independent CaM-binding protein that binds to CaM5 via its IQ motif. **(A)** Yeast two-hybrid assays of IQM4 and CaM5. The ORF of CaM5 was fused with the DNA binding domain in the bait vector pGBKT7 (BD-CaM5), and the ORF of IQM4 was fused with the activation domain in the prey vector pGADT7 (AD-IQM4). Yeast strain AH109 co-transformed with the AD-IQM4 and BD-CaM5 constructs could grow on the minimal synthetic dropout medium lacking Trp and Leu (SD-2), as well as that lacking Trp, Leu, His, and Ade (SD-4). BD-53 and AD-T comprised the positive control. AD and BD-CaM5 or AD-IQM4 and BD comprised the negative controls. **(B)** The IQ motif of IQM4 is required for the interaction between IQM4 and CaM5. Yeast strain AH109 harboring the AD-IQM4^Δ143-144^ (IQM4 with deletions of Leu^143^ and Gln^144^) and BD-CaM5 constructs as well as AD-IQM4^L143N^ (IQM4 with Leu^143^ substituted by Asn) and BD-CaM5 could not grow on SD-4. **(C)** Binding experiment between a truncated IQM4 (amino acids 1–160) and CaM5 *in vitro*. Fifty micrograms of crude separated protein from induced bacterial cells harboring an empty plasmid (CK) and a plasmid containing a partial ORF for the truncated IQM4 was transferred to nitrocellulose membranes and probed with biotinylated CaM5 in the presence of 1 mM CaCl_2_ or 5 mM EGTA. **(D)** Bimolecular fluorescence complementation (BiFC) detection of IQM4–CaM5 interactions in onion epidermal cells; nEYFP-IQM4 and cEYFP, nEYFP-IQM4 and cEYFP-CaM5, and nEYFP and cEYFP-CaM5 were bombarded into onion epidermal cells from left to right. Fluorescence images (top) and bright-field images (bottom) were obtained with epifluorescence microscopy. **(E)** BiFC detection of IQM4–CaM5 interactions in *Arabidopsis* mesophyll protoplast; nEYFP-IQM4 and cEYFP-CaM5, nEYFP and cEYFP-CaM5, and nEYFP-IQM4 and cEYFP were introduced into protoplast from up to down. Bright, bright-field image; YFP, YFP fluorescence image; Auto, chloroplast fluorescence; Merged, merged bright-field, YFP fluorescence, and chloroplast fluorescence images, scale bar: 10 μm.

Previously, we identified a novel IQ motif-containing CaMBP family using bioinformatics methods (Zhou et al., 2010) and verified that *IQM1* encodes a Ca^2+^-independent CaMBP according to yeast two-hybrid, CaM gel overlay, and BiFC assays (Zhou et al., 2012). In this study, we identified IQM4 as another novel CaMBP using yeast two-hybrid, CaM gel overlay, and BiFC assays. *Arabidopsis* CaM2/3/5 is used as a canonical CaM in most CaM-binding assays (Moon et al., 2005), so CaM5 (encoded by At2g27030) was employed as the target CaM in these experiments. Yeasts that harbored both AD-IQM4 and BD-CaM5 could grow on minimal SD medium lacking Trp, Leu, His, and Ade (SD-4) in a similar manner to the positive control (**Figure 1A**), i.e., IQM4 could bind with CaM5 in yeast cells.

The IQ motif present in many of the known Ca^2+^-independent CaMBPs contains the consensus sequence, IQxxxRGxxxR (Bähler and Rhoads, 2002). Previous studies have shown that the IQ motif is required for Ca^2+^-independent CaM complex formation and single amino acid changes in this motif, such as the first two amino acids “IQ,” abrogate both AtBAG6-activated CaM-binding and cell death in yeasts and plants (Kang et al., 2006). The N-terminal half of IQM4 contains the consensus sequence (amino acids 135–157) of the IQ motif. In order to identify the crucial amino acid residues in the IQ motif for IQM4-CaM5 complex formation, we employed two approaches for site-directed mutation of the IQ motif, where we deleted L^143^Q^144^ (designated as IQM4^Δ143-144^) and substituted Leu^143^ with Asn^143^ (designated as IQM4^L143N^). Interestingly, the results showed that both IQM4^Δ143-144^ and IQM4^L143N^ could not bind to CaM5 (**Figure 1B**), thereby demonstrating that IQM4 binds to CaM5 via these amino acid residues.

In most cases, proteins containing the IQ motif can interact with CaM in the absence of Ca^2+^ *in vitro* (Bähler and Rhoads, 2002). We conducted a CaM gel overlay assay to investigate whether IQM4 binding with CaM5 requires Ca^2+^. His-tagged CaM5 and an IQM4 truncate (residues 1–160) that encompassed a putative IQ motif (residues 135–157) were expressed in *Escherichia coli* and purified using affinity chromatography (**Supplementary Figures S2A–C**). The purified IQM4 truncate was separated with SDS-PAGE, blotted onto a membrane, and then overlaid with CaM5 labeled with biotin (**Supplementary Figures S2D,E**). As shown in **Figure 1C**, the IQM4 truncation could bind with CaM5 irrespective of the presence of Ca^2+^, thereby suggesting that IQM4 was a Ca^2+^-independent CaMBP *in vitro*.

The BiFC assay was performed in onion epidermal cells and *Arabidopsis* mesophyll protoplasts to further test whether IQM4 can bind with CaM5 in plant cells (**Figure 1D**). IQM4 was fused to the C-terminus of nEYFP and CaM5 was fused to the C-terminus of cEYFP. Both were driven by a cauliflower mosaic virus (CaMV) 35S promoter. The results showed that GFP fluorescence has been observed in 22 cells of about 50 intact onion cells in three replications when the nEYFP-IQM4 and cEYFP-CaM5 constructs were expressed in the onion cells, but the others combination didn’t be observed any fluorescence in all observed cells. In addition, YFP fluorescence has been observed in eight cells when the nEYFP-IQM4 and cEYFP-CaM5 constructs were expressed in *Arabidopsis* mesophyll protoplasts, but for the other combinations fluorescence was not detected. The observed pattern of YFP fluorescence suggests that the interaction between IQM4 and CaM5 may occur in the chloroplast (**Figure 1E**).

Overall, these results indicate that IQM4 can bind with CaM5 *in vitro* and *in vivo*, and that the interaction is a Ca^2+^-independent manner.

### Subcellular Localization of IQM4 Protein

**FIGURE 2:**
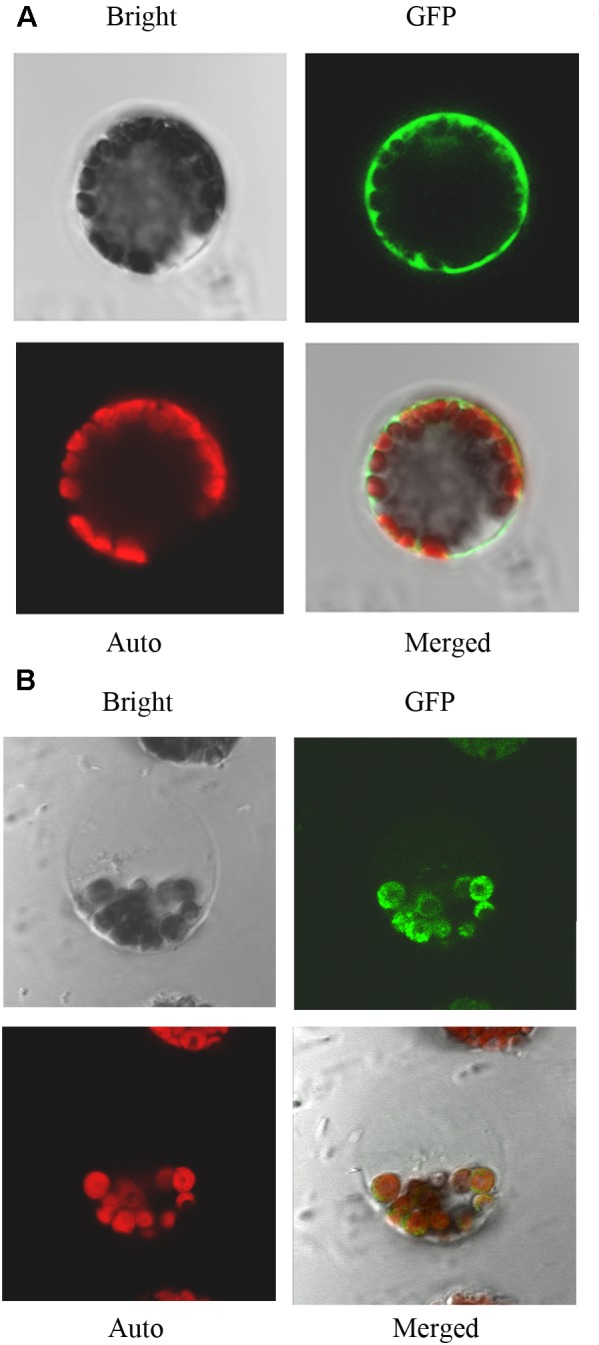
Subcellular localization of IQM4 protein. **(A)** Localization of GFP in *Arabidopsis* mesophyll protoplast. **(B)** Localization of IQM4-GFP fusion protein in *Arabidopsis* mesophyll protoplast. Bright, bright-field image; GFP, GFP fluorescence image; Auto, chloroplast fluorescence; Merged, merged bright-field, GFP fluorescence, and chloroplast fluorescence images.

Using network resources, several software tools including TargetP, ChloroP, and Predotar have predicted that the subcellular localization of IQM4 is in plastids^2^. Thus, in order to investigate the subcellular localization of IQM4 protein, both the control vector pIQM4::GFP and fusion gene vector pIQM4::IQM4-GFP were constructed, and transformed into *Arabidopsis* mesophyll protoplast using a PEG-mediated method (Sheen, 2001). GFP fluorescence was observed by confocal laser-scanning microscopy after incubation for 60 h at 23°C. The results showed that the GFP protein was localized in the cytosol (**Figure 2A**), whereas the IQM4-GFP fusion protein was localized exclusively in the chloroplast (**Figure 2B**), thereby indicating that the IQM4 protein is localized in the plastids and chloroplasts in plants. However, it is still unclear whether the IQM4 protein is localized in the outer membrane of chloroplast.

### Expression Profile of *IQM4*

**FIGURE 3:**
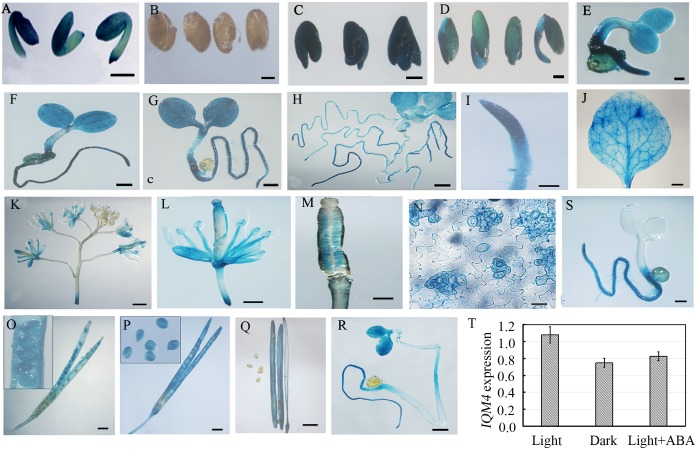
Histochemical GUS staining in different tissues of pIQM4:GUS transgenic plants. **(A)** Seed embryo. **(B)** Testa. **(C)** Embryo imbibed for 1 day. **(D–H)** Light grown seedlings aged 2, 3, 4, 5, and 10 days. **(I)** Root from 5-day-old seedling. **(J)** Mature leaf. **(K)** Inflorescence. **(L)** Flower. **(M)** Stigma. **(N**) Leaf epidermis. **(O–Q)** Developing embryo and silique at 5, 10, and 15 days after pollination (DAP). **(R)** Dark-grown seedling aged 5 days. **(S)** Light-grown seedling aged 5 days in 1/2 MS medium with 0.5 μM ABA. **(T)** *IQM4* expression level in dark-grown seedling and by ABA treatment. Scale bars: **(A–D,R,S)** 300 μm; **(I,M,N)** 150 μm; **(E–H,J–M)** 500 μm; **(O–Q)** 1 mm.

To understand the *IQM4* expression profile in greater detail, we fused the putative promoter region of the *IQM4* gene to the GUS reporter and analyzed the GUS activity in transgenic lines using a histochemical procedure (Jefferson et al., 1987). The *IQM4* promoter was highly active in the embryos of dry and imbibed seeds (stratified for 3 days) (**Figures 3A,C**), but not in the testa (**Figure 3B**). GUS activity was also detected in most of the tissues of the young seedlings (**Figures 3D–H**), where the highest GUS activity was in the proximal root (**Figure 3I**). *IQM4* was also expressed in the rosette leaf, flower, and silique (**Figures 3J–M,O–Q**), but especially in the guard cells in the epidermis (**Figure 3N**). The filament and stigma of the flower, and the developing embryo in green siliques also had higher GUS activity levels (**Figures 3L,M,O–Q**). The GUS staining in 5-day-old light-grown seedlings (**Figure 3G**) was slightly darker than that of dark-grown seedlings (**Figure 3R**), whereas the real-time RT-PCR (qRT-PCR) showed that there wasn’t significant difference in the levels of *IQM4* transcripts in both light- and dark-grown seedlings (**Figure 3T**). Intriguingly, ABA inhibited the expression of *IQM4* in the cotyledons and hypocotyl, but not in the root (compare **Figure 3S** and **Figure 3G**), and the qRT-PCR indicated that ABA didn’t significantly reduce the *IQM4* expression level (**Figure 3T**). Therefore, these results indicate that *IQM4* may be involved with seed development and germination, as well as ABA signaling.

### Isolation of T-DNA Insertion Lines of IQM4

To elucidate the functions of *IQM4* in plant growth and development, two T-DNA insertion alleles of *IQM4* in *Arabidopsis* were obtained from the ABRC. Two insertion positions in the *IQM4* gene were identified by PCR using F1, R1, and LBb1 primers (**Supplementary Figures S1B,C and Supplementary Table S1**). Sequencing of the genomic DNA flanking the T-DNA insertion showed that one insertion site of T-DNA was located in the sixth intron (*iqm4-1*, SALK_101916) and the other insertion site of T-DNA was located in the ninth exon, which was 86 bp downstream of the termination codon (*iqm4-2*, SALK_120435) (Supplementary Figure S1A). *IQM4* mRNA could not be detected in the *iqm4-1* mutant, but the mRNA could be detected by RT-PCR in the *iqm4-2* mutant using the F1 primer in the second exon (F1 in **Supplementary Figure S1A**) and the R1 primer upstream of the termination codon (R1 in **Supplementary Figures S1A,D**). However, *IQM4* mRNA could not be detected in both the *iqm4-1* and *iqm4-2* mutants when RT-qPCR was performed using the F2 and R2 primers (**Supplementary Figure S1E and Supplementary Table S1**), thereby indicating that both *iqm4-1* and *iqm4-2* could be used as the mutants of *IQM4*.

### T-DNA Insertion in *IQM4* Increases Primary Seed Dormancy

**FIGURE 4:**
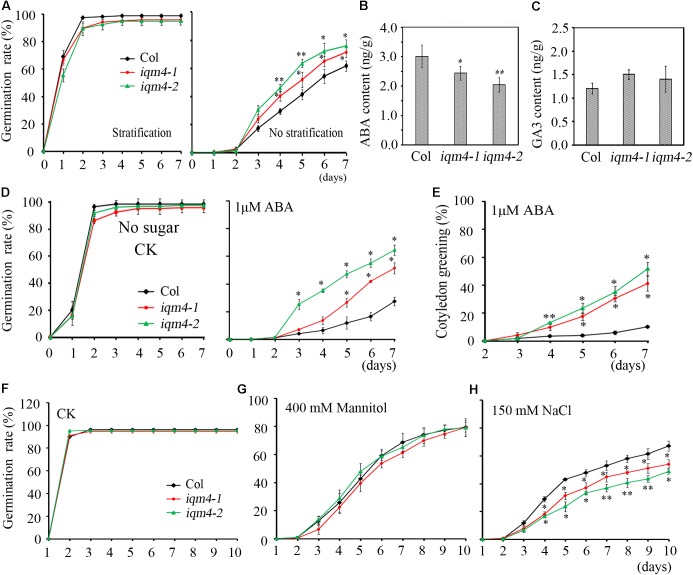
Primary seed dormancy, ABA content, and sensitivity of *iqm4* during seed germination. **(A)** Time-course of the germination rates by wild type, *iqm4-1*, and *iqm4-2* seeds on 1/2 MS medium with or without stratification treatment. Dry seeds stored for 1 week were used in **A–C**. **(B)** Endogenous ABA contents. **(C)** Endogenous GA3 contents. **(D)** Effects of exogenous ABA on seed germination by the wild type, *iqm4-1*, and *iqm4-2* mutants in 1/2 MS medium without 1% sucrose. The germination rate (full emergence of radicle) was scored daily after transfer to 23°C. **(E)** Effects of exogenous ABA on cotyledon greening (seedlings with green cotyledons) when grown on 1/2 MS medium without 1% sucrose. **(F)** Time-course of the germination rate by the wild type, *iqm4-1*, and *iqm4-2* seeds on 1/2 MS medium supplemented with 400 mM Mannitol **(G)** and 150 mM NaCl **(H)**. All data are shown as the mean ± SE (*n* = 4), and they were analyzed using the Student’s *t-*test, where the threshold of significance is indicated above (^∗^*P* < 0.05; ^∗∗^*P* < 0.01). Wild type (Col) in each group was used as a control.

The seed dormancy status increases during seed maturation and reaches a maximum in seeds at the harvest-time (Karssen et al., 1983). During the subsequent dry storage of seeds (after ripening), the dormancy status reduces until seeds are able to complete germination when imbibed under favorable conditions (Holdsworth et al., 2008). In the present study, the seed germination assay was performed using fresh seeds that underwent dry storage for 1 week. When all of the seeds were stratified for 3 days, both *iqm4* mutants and wild type seeds had the same germination rate (**Figure 4A**, left), but *iqm4-1* and *iqm4-2* had distinctly higher germination rates than wild type seeds without stratification treatment (**Figure 4A**, right). Thus, mutation of *IQM4* reduced the primary seed dormancy.

### T-DNA Insertion in *IQM4* Decreases the ABA Content of Dry Seeds

The ABA/GA ratio determines the fate of a seed, where a high endogenous ABA level and low GA level result in deep dormancy and low radical emergence; whereas a low ABA level and high GA level induce pre-harvest sprouting (Finkelstein et al., 2008; Shu et al., 2016). To understand the role of *IQM4* in seed dormancy and germination, the endogenous ABA and GA3 content of dry seeds were measured using a liquid chromatography-electrospray ionization tandem mass spectrometry system. Seeds that underwent dry storage for 1 week after harvesting were used in this experiment; the ABA content of dry *iqm4* seeds was significantly lower than that of the wild type, with the *iqm4-2* mutant showing the lowest content (**Figure 4B**). Whereas, the GA3 content of dry *iqm4* seeds was comparable to that of wild type (**Figure 4C**). These results indicate that mutation of *IQM4* affected the endogenous ABA level, which may partly explain why *iqm4* seeds exhibited reduced primary seed dormancy.

### T-DNA Insertion in *IQM4* Reduces the Sensitivity to ABA During Seed Germination and Seedling Growth

Exogenous ABA inhibits seed germination and post-germination seedling growth (Finkelstein et al., 2002, 2008). In previous studies, multiple mutants such as *abi1-1, abi2-1, abi3*, and *abi4-1* exhibited reduced seed dormancy and insensitivity to ABA during germination (Koornneef et al., 1984, 1989; Shu et al., 2013). Low concentrations of sugar (90 mM sucrose) can overcome ABA-inhibited radicle emergence, but greening and subsequent seedling growth are still blocked (Finkelstein and Lynch, 2000). To investigate the effects of *IQM4* mutation on the ABA-induced inhibition of seed germination, wild type and *iqm4* seeds with stratification treatment were sown on MS medium containing different concentrations of ABA with or without sucrose. Similar results were obtained irrespective of whether sucrose was added or not added to the medium. The germination rates of *iqm4-1* and *iqm4-2* were similar to that of wild type seeds under control conditions (**Figure 4D** and **Supplementary Figure S3**), where exogenous ABA-induced inhibition of germination in *iqm4* mutants was significantly lower than that of wild type seeds with or without sugar, although sugar obviously reduced the effect of ABA inhibition on seed germination (**Figure 4D** and **Supplementary Figure S3**). In addition, the cotyledon greening rates were higher in the seedlings of *iqm4-1* and *iqm4-2* mutants than in wild type seedlings (**Figure 4E**). The results showed that mutation of *IQM4* decreased the sensitivity to ABA during seed germination and seedling development.

**FIGURE 5:**
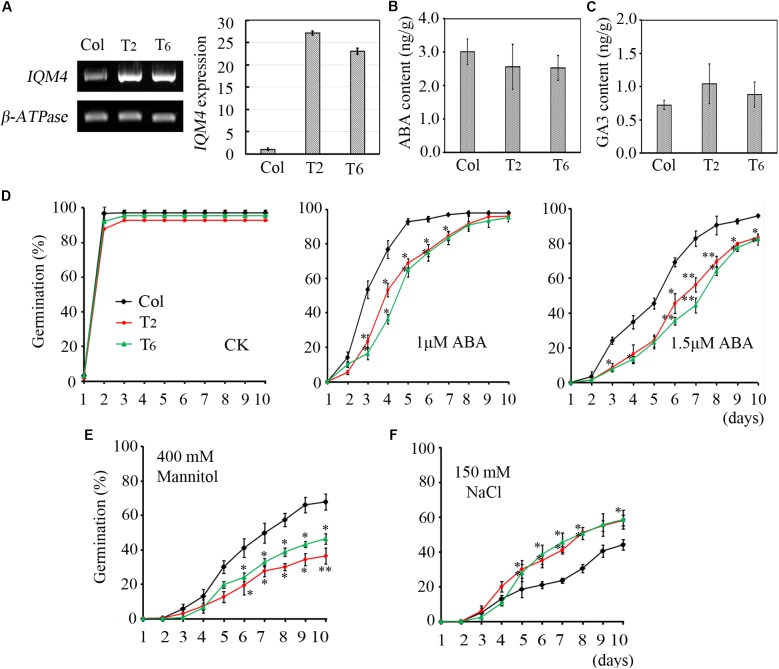
Effects of ABA, NaCl, and mannitol on seed germination in *IQM4* overexpression lines. **(A)** Two *IQM4* overexpression transgenic lines (T2 and T6) were identified using both RT-PCR and qRT-PCR. **(B)** Endogenous ABA contents. **(C)** Endogenous GA3 contents. **(D)** Time-course of germination rate by the wild type, T2, and T6 on 1/2 MS medium without (CK) or with 1 or 1.5 μM ABA. **(E)** Time-course of the germination rate by the wild type, T2, and T6 on 1/2MS medium with 400 mM mannitol. **(F)** Time-course of the germination rate by the wild type, T2, and T6 on 1/2 MS medium with 150 mM NaCl. All data are shown as the mean ± SE (*n* = 4), and they were analyzed using the Student’s *t*-test, where the threshold of significance is indicated above (^∗^*P* < 0.05; ^∗∗^*P* < 0.01). Wild type (Col) in each group was used as a control.

To further investigate whether *IQM4* is involved with the ABA-induced inhibition of primary root growth, 5-day-old wild type and *iqm4* seedlings were transferred to media with or without ABA and grown for 7 days. The results showed that *iqm4* seedlings had shorter primary roots than wild type under control conditions, where the primary root length of all seedlings was similar in the presence of 3 μM ABA, but the primary root length of *iqm4* seedlings was significantly longer than that of wild type in the presence of 6 μM ABA (**Figure 5**). These results suggest that mutation of *IQM4* reduced the primary root elongation and the sensitivity to ABA during seedling growth.

**FIGURE 6:**
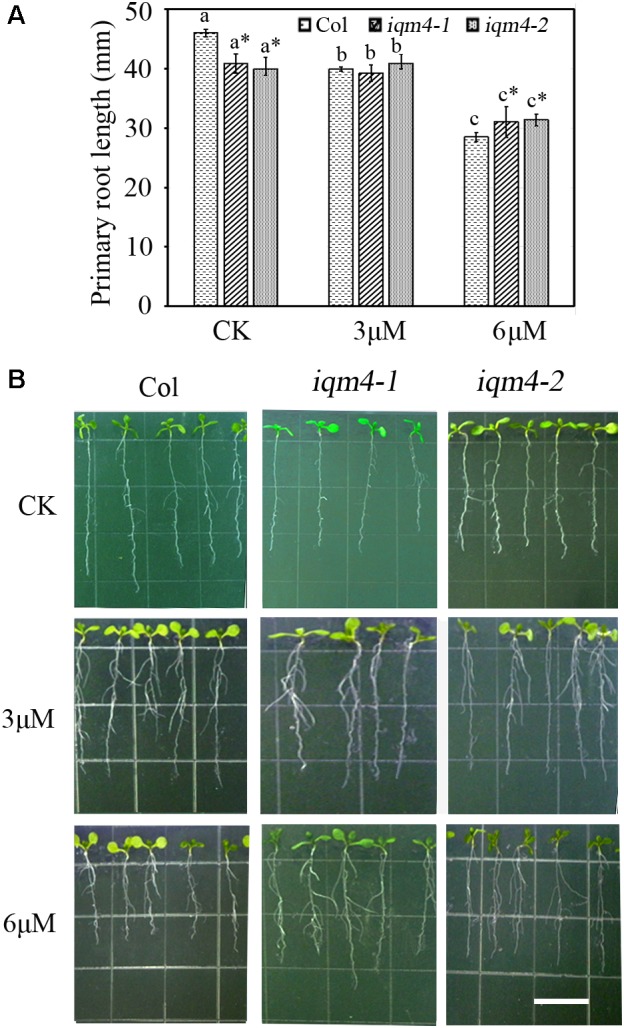
Effects of ABA on primary root length in *iqm4* seedlings. **(A)** Primary root length in the wild type, *iqm4-1*, and *iqm4-2* seedlings grown on 1/2 MS with 0, 3, or 6 μM ABA. Seedlings were grown vertically for 5 days, transferred to 1/2 MS medium with or without ABA, and vertically growth was continued for 7 days. Primary root length was measured using Scion Image software. All data are shown as the mean ± SE (*n* = 4) and they were analyzed using the Student’s *t*-test, where the threshold of significance is indicated above (^∗^*P* < 0.05; ^∗∗^*P* < 0.01). Wild type (Col) in each group was used as a control. **(B)** Representative images of 12-day-old seedlings with or without ABA. Scale bar: 15 mm.

### T-DNA Insertion in *IQM4* Increases the Sensitivity to Salt Stress

Abscisic acid plays essential roles in the tolerance of salt and osmotic stress, where these stress signals promote the expression of ABA biosynthesis genes, thereby leading to the accumulation of ABA (Leung and Giraudat, 1998). We showed that mutation of *IQM4* modulated the ABA level and the sensitivity to ABA in the seed (**Figures 4B,D**); therefore, *iqm4* may exhibit a specific phenotype under high-salt and hyperosmotic stress. As shown in **Figures 4G,H**, the germination of all genotypes was inhibited under mannitol and NaCl treatment, where *iqm4* and wild type seeds had a similar germination rate in response to mannitol stress (**Figure 4G**), but *iqm4* mutants had a lower germination rate than wild type seeds under NaCl treatment (**Figure 4H**). Thus, mutation of *IQM4* did not change the sensitivity to hyperosmotic stress but it increased the sensitivity to salt stress.

### *IQM4* Overexpression Enhances the Sensitivity to ABA and Osmotic Stress, and Reduces the Sensitivity to Salt Stress During Germination

To further explore the functions of *IQM4*, we constructed the p35S:IQM4 vector and obtained six independent homozygous T3 transgenic lines. RT-PCR and qRT-PCR results showed that the expression of *IQM4* was significantly elevated in the p35S:IQM4 transgenic lines (T2 and T6) (**Figure 5A**). Thus, T2 and T6 were used in subsequent assays. The ABA and GA3 contents of dry T2 and T6 seeds underwent dry storage for 1 week after harvesting were also measured. Unexpectedly, the ABA and GA3 contents of dry T2 and T6 seeds were similar to those of the wild type (**Figures 5B,C**). On MS medium, the germination rates of wild type, T2, and T6 seeds did not differ greatly, and the germination rates of T2 and T6 seeds were significantly lower than those of the wild type with 1.0 and 1.5 μM ABA (**Figure 5D**), whereas they were significantly higher under NaCl stress (**Figure 5F**). Interestingly, the germination rates of T2 and T6 seeds were distinctly lower than those of the wild type under mannitol stress (**Figure 5E**). Thus, the *IQM4*-overexpressing lines had lower germination rates under treatments with ABA and osmotic stress compared with wild type seeds, but had higher germination rates under salt stress.

As mentioned above, the sensitivity to ABA during germination was enhanced by *IQM4* overexpression but was decreased by the T-DNA insertion in *IQM4*; this result was the opposite of that obtained under salt stress. However, only overexpression of *IQM4* decreased the germination rate under hyperosmotic stress compared with the wild type. These results suggest that *IQM4* was involved with ABA, salt, and osmotic stress response during seed germination.

### Effect of *IQM4* Mutation on the Transcription of ABA Metabolism Genes and Seed Maturation Regulators in Developing Siliques

**FIGURE 7:**
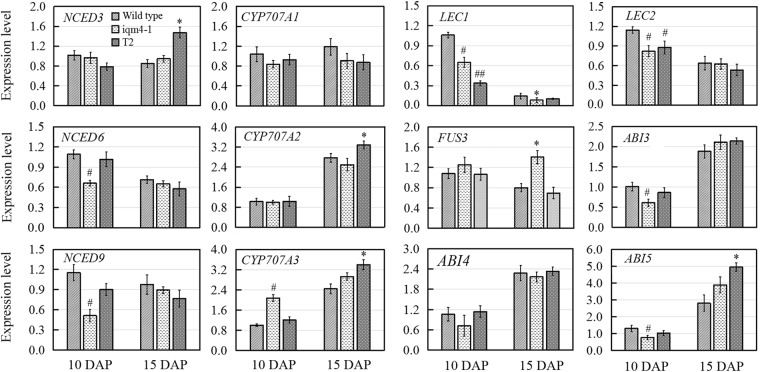
Effects of *IQM4* mutation on the transcript levels of embryo development regulators and major ABA metabolism genes in developing siliques. Total RNA was extracted from siliques of the wild type (Col), *iqm4-1*, and *IQM4*-overexpressing line (T2) at 10 and 15 days after pollination (DAP). Transcript levels were examined by real-time RT-PCR using the expression of the *ACTIN2* housekeeping gene as an internal control. Four independent experiments were performed. All of the data are shown as the mean ± SE (*n* = 4), and they were analyzed using the Student’s *t*-test, where # and ^∗^ indicate significant differences compared with wild type siliques at 10 DAP and at 15 DAP, respectively; ^#^ or ^∗^*P* < 0.05; ^##^ or ^∗∗^*P* < 0.01.

There are two ABA accumulation peaks during seed maturation in *Arabidopsis*, where the first peak is maternally derived and it precedes the seed maturation phase, and the second peak depends on synthesis in the embryo itself (Karssen et al., 1983). Embryonic ABA is essential for inducing and maintaining seed dormancy, and thus preventing vivipary (Finkelstein et al., 2002). To determine whether the lower ABA level in *iqm4* seeds were correlated with the transcription of ABA metabolism genes, qRT-PCR was performed in immature siliques at 10 and 15 days after pollination (DAP). The results showed that the expression levels of both *NCED6* and *NCED9* were distinctly decreased in the developing siliques of *iqm4-1* at 10 DAP, whereas those of *NCED3* were significantly increased in T2 at 15 DAP (**Figure 7**). In addition, *CYP707A3* transcription was significantly increased in the developing siliques of *iqm4-1* at 10 DAP, and *CYP707A2-A3* expression levels were also increased in T2 at 15 DAP (**Figure 7**). It is well known that the endogenous ABA level is regulated through the dynamic balancing of ABA biosynthesis and the catabolism pathway, which includes the feedback induction of catabolism (Nambara and Marion-Poll, 2005; Nonogaki et al., 2014). Therefore, this may explain why the T-DNA insertion in *IQM4* inhibited *NCED6* and *NCED9* expression in the developing siliques, thereby leading to lower ABA content in *iqm4* seeds. However, the overexpression of *IQM4* enhanced the expression of ABA biosynthesis genes (*NCED3*) and catabolism genes (*CYP707A2* and *A3*) at 15 DAP, which may partly explain why both T2 and T6 had similar ABA content to the wild type. Clearly, underlying molecular mechanisms involved in these processes need to be explored further.

*LAFL* genes are embryo developmental regulators that have distinct temporal expression patterns during seed development (Jia et al., 2013). Previous studies have shown that the maximum expression levels of *LEC1* and *LEC2* occur in the heart embryo stage; the expression of *FUS3* peaks during early seed maturation; whereas *ABI3* is expressed throughout the maturation phase (To et al., 2006; Jia et al., 2013). Moreover, ABI4 plays a role in primary seed dormancy by regulating ABA and GA homeostasis (Shu et al., 2013). ABI5 is involved in seed maturation and ABA signaling in vegetative tissues (Finkelstein and Lynch, 2000). Thus, multiple seed development regulators were examined in this study in order to investigate the effects of *IQM4* on the expression of embryo development regulators. As shown in **Figure 7**, *LEC1, LEC2, ABI3*, and *ABI5* transcript levels were significantly reduced in the developing siliques of *iqm4-1* at 10 DAP, while the expression levels of *LEC1* and *LEC2* were also decreased in T2 at 10 DAP. The expression of *FUS3* was enhanced in the developing siliques of *iqm4-1*, as was the expression levels of *ABI5* in T2 at 15 DAP. Thus, the mutation of *IQM4* affected the transcript levels of all the *LAFL* genes. In addition, overexpression of *IQM4* enhanced the expression levels of ABA signaling genes (*ABI5*) during late maturation.

### Effects of *IQM4* Mutation on the Transcript Levels of ABA Signaling Genes During Germination

In *Arabidopsis*, the PP2Cs comprise ABI1, ABI2, HAB1, HAB2, AHG1, and PP2CA/AGH3, which have been shown to interact directly with the ABA receptor, PYR/PYL/RCAR (Schweighofer et al., 2004; Ma et al., 2009; Park et al., 2009). *Arabidopsis* SnRK2 protein kinases (SnRK2.2/2.3/2.6) were identified as key positive regulators of ABA signaling, phosphorylation, and as activators of the downstream bZIP transcription factor ABI5, as well as regulators of ABA-responsive genes that mediate ABA signaling (Fujii et al., 2007; Fujii and Zhu, 2009). Three transcription factors comprising *ABI3, ABI4*, and *ABI5* are all positive regulators in ABA signaling and their expression is induced by exogenous ABA. Unlike ABI transcription factors, WRKY40 functions as a negative regulator and its expression is inhibited by ABA, thereby directly regulating a set of ABA-responsive genes including *ABI4, ABI5, ABF4*, and *MYB2*(Shang et al., 2010; Liu et al., 2012).

**FIGURE 8:**
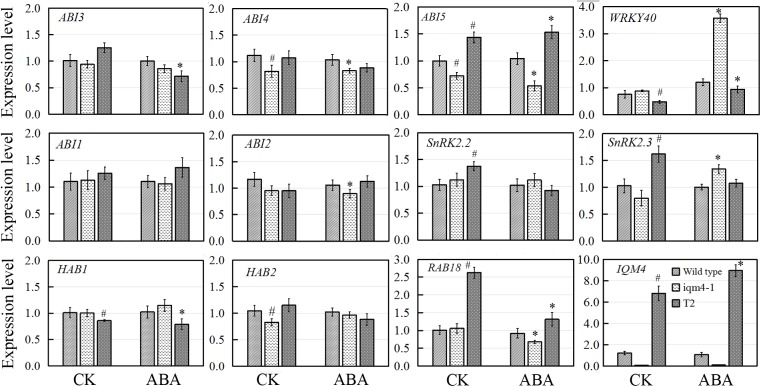
Effects of *IQM4* mutation on the transcript levels of key ABA signaling genes during seed germination. Total RNA was extracted from seedlings of the wild type (Col), *iqm4-1*, and *IQM4*-overexpressing line (T2) grown on 1/2 MS medium without (CK) or with 3 μM ABA (ABA) for 2 days following stratification. Transcript levels were determined by real-time RT-PCR using the expression level of the *ACTIN2* housekeeping gene as an internal control. Four independent experiments were performed. All of the data are shown as the mean ± SE (*n* = 4), and they were analyzed using the Student’s *t*-test, where # and ^∗^ indicate significant differences compared with wild type (CK) and wild type (ABA) seeds, respectively; ^∗^ or ^#^*P* < 0.05; ^∗∗^or ^##^*P* < 0.01.

To elucidate how *IQM4* mediates ABA signaling, we analyzed the expression of ABA signaling genes in *iqm4-1* mutants and an *IQM4*-overexpressing line (T2) during ABA-induced inhibition of seed germination. For confirming the genotypes of wild type (Col), *iqm4-1* mutant and *IQM4*-overexpressing line (T2), the expression level of *IQM4* was analyzed, the result showed that the expression of *IQM4* was almost abolished in *iqm4-1* mutant whereas it was significantly up-regulated in the T2 line (**Figure 8**). Under control conditions, the expression levels of *ABI4, ABI5*, and *HAB2* in *iqm4-1* seedlings and those of *WRKY40* and *HAB1* in the T2 line were down-regulated. Those of *ABI5, SnRK2.2, SnRK2.3*, and *RAB18* were up-regulated in the T2 line. When all of the seeds were sown on MS in the presence of ABA, the expression of *ABI4, ABI5, ABI2*, and *RAB18* were decreased whereas those of *WRKY40* and SnRK2.3 were increased in *iqm4-1* mutant. The expression of *WRKY40* and *HAB1* were inhibited whereas those of *ABI5* and *RAB18* were increased in T2 line (**Figure 8**).

Overall, these results showed that the mutation of *IQM4* increased the expression of *WRKY40* but decreased that of *ABI4* and *ABI5* in response to ABA, while the expression levels of negative regulators such as *ABI2* and *HAB2*, as well as that of positive regulator such as *SnRK2.2* were also repressed in the *iqm4* mutant. According to the transcription levels of all genes analyzed in this study, *IQM4* is involved with the ABA regulation network as a positive regulator, but the molecular mechanisms that underlie its role in this regulation need to be studied further.

## Discussion

In this study, we confirm that *Arabidopsis* IQM4 is a novel Ca^2+^-independent CaMBP (**Figure 1**) that is localized in the chloroplasts of plant mesophyll cells (**Figure 2**). We conducted the expression and functional analyses of *IQM4* during seed development and germination to elucidate its roles in plant growth and development. Our results indicate that *IQM4* is implicated in seed dormancy and germination by modulating the endogenous ABA level and the sensitivity to ABA in seeds (**Figures 4–6**). Furthermore, qRT-PCR demonstrates that *IQM4* promotes ABA biosynthesis by regulating the expression of both *NCED6* and *NCED9* in developing siliques (**Figure 7**), and *IQM4* is also involved in ABA signaling by repressing the expression of *WRKY40* during seed germination and post-germination growth (**Figure 8**). Thus, we suggest that *IQM4* is a positive regulator of ABA responses during seed dormancy, germination, and seedling growth.

### *IQM4* Encodes a Novel Ca^2+^-Independent CaMBP

Several CaMBPs have been identified using traditional approaches such as yeast two-hybrid assays, expression library screening with labeled CaM probes, and CaM overlay assays, and many proteins have been predicted to bind with CaMs based on their structural homology with known targets (Popescu et al., 2007; Reddy et al., 2011). As the plant BiFC assay is a simple, reliable, and relatively fast method for determining protein–protein interactions in plants (Bracha-Drori et al., 2004), we used yeast two-hybrid and BiFC assays to demonstrated that IQM4 can bind to canonic CaM5 *in vivo* (**Figures 1A,D**). The N-terminal half of IQM4 contains a consensus sequence for the IQ motif (LQKVYKSYRTR), which is present in many of the known Ca^2+^-independent CaMBPs (Bähler and Rhoads, 2002), and thus we investigated whether IQM4 interacts with CaM5 via the IQ motif and in a Ca^2+^-independent manner. L^143^Q^144^ in the IQ motif (amino acids 136–165) was deleted (IQM4^Δ143-144^) or Leu^143^ was substituted with Asn (IQM4^L143N^), and the results suggested that the IQ motif is essential for IQM4-CaM5 complex formation in yeast cells (**Figure 1B**). In addition, a CaM overlay assay was conducted to verify IQM4 binding in a Ca^2+^-independent manner, as shown in **Figure 1C**, where we found that IQM4 can bind with CaM5 in the presence of CaCl_2_ and EGTA. Thus, IQM4 was identified as a novel Ca^2+^-independent CaMBP in the *Arabidopsis* IQM family. In addition, while CaM5 is able to interact with IQM4 in onion epidermal cells and *Arabidopsis* mesophyll protoplasts (**Figures 1D,E**), and IQM4 is localized in the chloroplasts (**Figure 2**), the interaction of IQM4 and CaM5 also occurs in the chloroplasts of mesophyll cell.

### *IQM*4 Positively Regulates Primary Seed Dormancy

In this study, *IQM4* was expressed in most tissues, especially the germinated embryo and the root tip (**Figure 3**), and darkness and exogenous ABA application didn’t significantly alter the *IQM4* expression level in 5-day-old seedlings (**Figures 3R–T**). These observations are consistent with the data obtained previously by RT-PCR (Zhou et al., 2010) and the microarray method^3^. The expression pattern can provide important insights into gene functions, so the biological relevance of the *IQM4* expression pattern was assessed using two T-DNA insertion mutants (*iqm4-1* and *iqm4-2*) (**Supplementary Figure S3**). The seed germination and hormone measurement assays demonstrated clearly that *iqm4* seeds had lower ABA content (**Figure 4B**), as well as reduced primary seed dormancy compared with wild type seeds, but that stratification abolished this difference (**Figure 4A**). ABA is an important positive regulator of seed dormancy induction and maintenance (Kucera et al., 2005; Finch-Savage and Leubner-Metzger, 2006). In *Arabidopsis*, there are two ABA accumulation peaks, wherein the first peak ABA is synthesized in maternal tissues during mid-maturation (at about 10 DAP) and in the second, where ABA is derived from the zygotic tissues during late maturation (at about 15 DAP), which is essential for the induction and maintenance of primary seed dormancy (Karssen et al., 1983; Koornneef et al., 1989; Raz et al., 2001). A previous study found that *AtNCED6* was expressed in the endosperm and *AtNCED9* was expressed in both the endosperm and the embryo, while ABA content in *nced6* and *nced9* seeds was lower than that in the wild type, with the *nced6nced9* double mutant exhibiting the reduced seed dormancy (Lefebvre et al., 2006). Microarray data demonstrated that the expression of *AtNCED5* increased in the late maturation stages, which was confirmed by analyses of developing seeds of pNCED5:GUS transgenic plants (Frey et al., 2012). In the present study, T-DNA insertion in *IQM4* distinctly decreased the transcript levels of *NCED6* and *NCED9* in developing siliques at 10 DAP, and the overexpression of *IQM4* significantly increased the transcript levels of *NCED3* in developing siliques at 15 DAP (**Figure 7**); it should be noted that the *NCED5* expression level was too low to be detected at 10 DAP in this experiment. These results suggest that *IQM4* plays a key role in promoting the expression of *NCED6* and *NCED9* during seed maturation.

The accumulation of ABA is determined by the precise dynamic balance between ABA biosynthesis and catabolism pathways, including feedback induction of catabolism (Nambara and Marion-Poll, 2005; Nonogaki et al., 2014). In plants, ABA 8′-hydroxylation is considered to play a predominant role in ABA catabolism. Members of the *CYP707A* family, i.e., *CYP707A1–CYP707A4*, encode ABA 8′-hydroxylases, which are the key enzymes in ABA catabolism (Kushiro et al., 2004). CYP707A1 and CYP707A2 are the major isoforms during mid-maturation and late maturation, respectively (Okamoto et al., 2006). In a previous study, fresh seeds of two *cyp707a2* mutants exhibited the reduced germination potential, and *cyp707a2* seeds accumulated six times as much ABA as wild-type seeds during imbibition (Kushiro et al., 2004). *CYP707A3* mainly contribute to ABA catabolism during post-germination growth, and there were no differences in the ABA levels in dry seeds of *cyp707a3* mutants and the wild type (Okamoto et al., 2006). In the present study, DNA insertion in *IQM4* did not alter the expression levels of both *CYP707A1* and *CYP707A2* in the developing siliques at 10 and 15 DAP, but it enhanced *CYP707A3* expression at 10 DAP (**Figure 7**). Considering the biosynthesis of ABA, we may conclude that *IQM4* promotes the ABA biosynthesis by enhancing the expression of the *NCED* genes during seed maturation. Overexpression of *IQM4* did not change the ABA levels in dry seeds, which may partly be explained by the overexpression of *IQM4* promoting the expression of the ABA biosynthesis gene (*NCED3*) as well as catabolism genes (*CYP707A2* and *CYP707A3*) during late maturation (**Figure 7**).

It is well established that the *LAFL* gene network comprising *LEC2, ABI3, FUS3*, and *LEC1* plays a central role in the integration of hormonal and intrinsic developmental signals that control embryo development (Holdsworth et al., 2008; Jia et al., 2014). Moreover, two well-characterized transcription factors, *ABI4* and *ABI5*, are positive regulators of seed maturation and negative regulators of seed germination, (Finkelstein et al., 1998; Finkelstein and Lynch, 2000). *ABI4* and *ABI5* are expressed predominantly in developing and mature seeds, and they are regulated by *ABI3* and ABA (Penfield et al., 2006; Reeves et al., 2011). Recently, it was reported that ABI4 plays a role in primary seed dormancy by regulating ABA and GA homeostasis (Shu et al., 2013). In the present study, T-DNA insertion in *IQM4* significantly influenced the transcript levels of all *LAFL* genes, where those of *LEC1, LEC2*, and *ABI3* decreased, but *FUS3* mRNA levels increased in the late maturation stage; whereas overexpression of *IQM4* increased the transcript level of *ABI5* in the late maturation stage (**Figure 7**). These results suggest that *IQM4* is involved with the regulation of seed maturation genes.

We conclude that *IQM4* affects the expression of ABA biosynthesis genes and seed maturation regulators, thereby regulating primary seed dormancy.

### *IQM4* Positively Regulates ABA Signaling in Germination and Post-germination Growth

It is widely considered that ABA prevents germination and inhibits seedling growth via the complex ABA signaling pathway (Finkelstein et al., 2002). Some abiotic stresses such as high salinity and drought can promote ABA production, where ABA acts through the signaling cascade to induce adaptive responses in multiple physiological processes (Xiong et al., 2002). In the present study, the *iqm4* mutants had ABA-insensitive phenotypes during germination and post-germination growth (**Figures 4D**, **6**), whereas the *IQM4*-overexpressing lines were ABA-hypersensitive (**Figure 5D**). These results demonstrate that *IQM4* is positively involved in ABA signaling during germination and post-germination growth. Moreover, *iqm4* mutants were salt-hypersensitive (**Figure 4H**) whereas the *IQM4*-overexpressing lines were salt-insensitive (**Figure 5F**). Under high-osmotic stress, *iqm4* mutants were similar to wild type seeds (**Figure 4G**) but the *IQM4*-overexpressing lines were more sensitive (**Figure 5E**). It looks like a contradiction that the germination rate of *iqm4* mutants was lower under salt stress (**Figure 4H**), and was higher under ABA treatment (**Figure 4D** and **Supplementary Figure S3**). However, the results of seed germination assays indicate that *IQM4* can promote seed germination under salt stress conditions (**Figures 4H**, **5F**), and repress seed germination under osmotic stress condition (**Figure 5E**). It is possible that there are many opening questions about the interaction of ABA signaling with salt/osmotic signaling.

Three transcription factors ABI3, ABI4, and ABI5 are well-known positive regulators of ABA signaling during seed germination and post-germination growth (Giraudat et al., 1992; Finkelstein et al., 1998; Finkelstein and Lynch, 2000). In the ABAR(ABA Receptor)-mediated ABA signaling pathway, ABAR/CHLH (H subunit of Mg-chelatase), a chloroplast/plastid protein, acts as an ABA receptor and relieves ABA responsive genes from inhibition by antagonizing the negative regulator, WRKY40, during seed germination and post-germination growth (Shen et al., 2006; Shang et al., 2010). *WRKY40* is a central negative regulator of ABA signaling and it inhibits the expression of a subset of ABA-responsive genes, including *ABI4, ABI5, ABF4, MYB2*, and *RAB18* (Shang et al., 2010; Liu et al., 2012). In the present study, overexpression of *IQM4* distinctly increased the expression of *ABI5* and *RAB18* but decreased the expression of *WRKY40* under the control condition and ABA treatment, whereas the T-DNA insertion in *IQM4* significantly decreased the expression of *ABI5* and *RAB18*, but increased the expression of *WRKY40* in the presence of high ABA level (**Figure 8**). In the ABA-ABAR-WRKY40-ABI5 signaling cascade, chloroplast ABAR relieves the *ABI5* gene from inhibition initiating translocation of WRKY40 from the nucleus to the cytosol to promote ABAR-WRKY40 interactions in response to a high concentration of ABA (Shen et al., 2006; Shang et al., 2010; Liu et al., 2012). In the present study, IQM4 protein localized in the chloroplast (**Figure 2**) to repress *WRKY40* expression and promote *ABI5* expression (**Figure 8**), therefore, it is possible that *IQM4* is involved in a retrograde signaling from the chloroplast.

However, the underlying molecular mechanisms involved need to be elucidated. It is well established that PP2Cs, including ABI1, ABI2, HAB1, and HAB2, are key negative regulators in ABA signaling (Ma et al., 2009; Park et al., 2009). The loss of function by *ABI1* and *ABI2* yields ABA-hypersensitive phenotypes and double mutation increases the hypersensitivity to ABA (Gosti et al., 1999; Merlot et al., 2001). In contrast, SnRK2s act as positive regulator by phosphorylating and activating downstream bZIP transcription factors such as ABI5 (Fujii et al., 2007; Fujii and Zhu, 2009). The *snrk2.2snrk2.3* double mutant exhibits high insensitivity to ABA-regulated germination compared with the wild type and single mutant (Fujii et al., 2007). In the present study, T-DNA insertion in *IQM4* reduced the expression of *ABI2* and *HAB2*, and *IQM4* overexpression significantly decreased the expression of *HAB1*, but increased the expression of *SnRK2.2* and *SnRK2.3* (**Figure 8**), which may be explained by the regulation role of *IQM4* in the ABA signaling and the feedback regulation of ABA signaling due to the low endogenous ABA level in *iqm4* seeds.

### Does IQM4 Mediate CaM Signaling During the Regulation of Seed Dormancy and/or Germination?

It is well known that the typical CaM acts as a Ca^2+^ relay by interacting with and regulating the activity of target proteins, which can participate in multiple cell physiology functions (Bouché et al., 2005). Previous studies have shown that several CaM/CMLs act as positive or negative regulators during the ABA-mediated inhibition of seed germination and post-germination growth, such as *AtCAM7* (Abbas and Chattopadhyay, 2014; Abbas et al., 2014), *AtCML9* (Magnan et al., 2008), *AtCML24* (Delk et al., 2005), *AtCML37* and *AtCML42* (Vadassery et al., 2012; Scholz et al., 2015), and *OsMSR2* (Xu et al., 2011). The downstream targets of these CaM/CMLs are mostly unknown, and how the specificity of Ca^2+^ signaling can be achieved through the actions of CaM/CMLs and their target proteins is also largely unknown. In the present study, we showed that *IQM4*, a novel Ca^2+^-independent CaMBP, positively regulates seed dormancy by promoting ABA biosynthesis and ABA signaling during seed maturation and germination. It is well established that a given Ca^2+^ signal might lead to different biochemical consequences in terms of its particular CaM/CML isoform-dependent target proteins. Therefore, the next challenge will entail identifying the upstream target proteins of IQM4 and characterizing their roles in ABA-regulated seed dormancy and germination. This research may lead to new insights into the crosstalk between ABA signaling and Ca^2+^-CaM signaling during plant development.

## Author Contributions

YPZ and C-ET designed the experiments. JHW, WHX, and WC performed the experiments. QHC, TF, CPX, and C-ET analyzed and discussed the results. YPZ, JHW, and C-ET wrote and revised the manuscript.

## Conflict of Interest Statement

The authors declare that the research was conducted in the absence of any commercial or financial relationships that could be construed as a potential conflict of interest.

## Footnotes

1 http://abrc.osu.edu/

2 http://suba.live/suba-app/factsheet.html?id=AT2G26190

3 http://www.genevestigator.com/gv/index.jsp
