# Supplementary material for: Arabidopsis IQM4, a Novel Calmodulin-Binding Protein, Is Involved With Seed Dormancy and Germination in Arabidopsis
Source: Front Plant Sci. 2018 Jun 5;9:721. doi: 10.3389/fpls.2018.00721 (PMC6008652; doi:10.3389/fpls.2018.00721)
Supplement: Supplementary file 1 [file Data_Sheet_1.PDF]

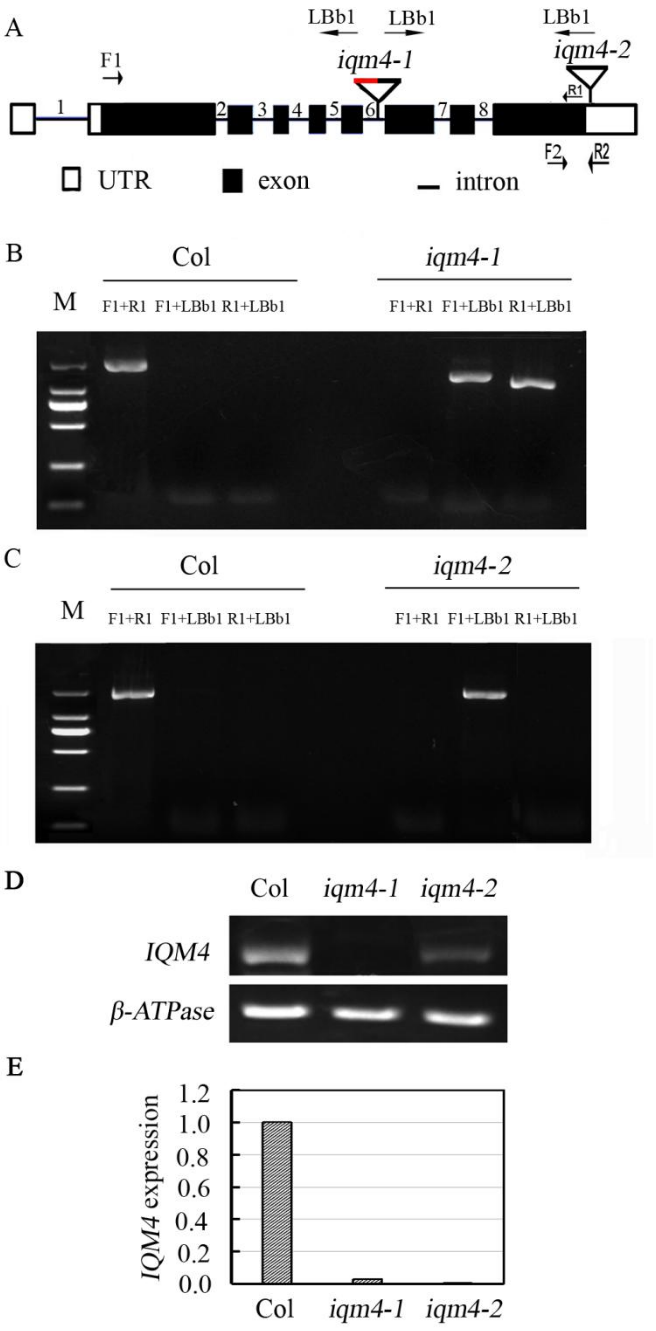

FIGURE S1 | Identification of T-DNA insertion sites in *iqm4-1* and *iqm4-2* by PCR. **(A)** Scheme for T-DNA insertion mutants. The intron and exon organization of the *IQM4* gene is depicted by lines and boxes, respectively. The positions of the T-DNA insertions are indicated by inverted triangles. The positions of the primers used for PCR, RT-PCR, and qRT-PCR are indicated by arrows. **(B)** Identification of *iqm4-1* mutant by PCR using P1, R1 and LBb1 primers. **(C)** Identification of *iqm4-2* mutant by PCR using P1, R1 and LBb1 primers.

(D) *IQM4* expression of *iqm4-1* and *iqm4-2* mutants were analyzed through RT-PCR (F1 and R1).

(E) *IQM4* expression of *iqm4-1* and *iqm4-2* mutants were analyzed through qRT-PCR (F2 and R2)

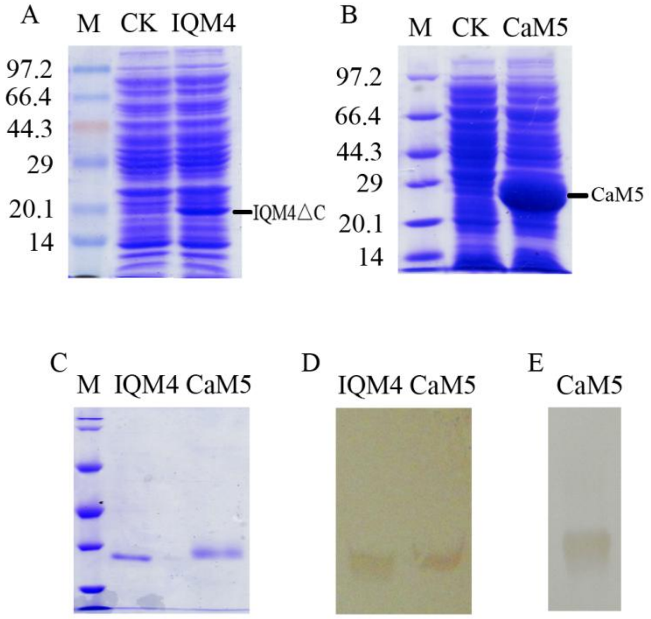

FIGURE S2 | Protein expression of the truncated IQM4 and CaM5 in bacterial cells and detection of the biotinylated CaM5. **(A)** The truncated IQM4 was separated with SDS-PAGE. **(B)** The CaM5 was separated with SDS-PAGE. **(C)** The purified his-tagged IQM4 and his-tagged CaM5 were detected with SDS-PAGE. **(D)** The his-tagged IQM4 and his-tagged CaM5 were detected by western blotting. **(E)** the biotinylated CaM5 was detected by western blotting.

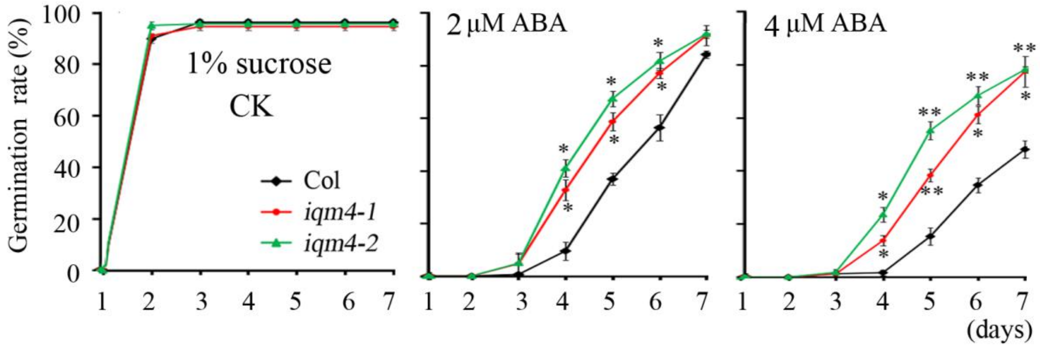

FIGURE S3 | Effects of different concentration of ABA on seed germination of WT and *iqm4* mutants in 1/2MS medium with 1% sucrose.

Data are shown as mean ± SE (n = 3). These data were analysed by the Student's t-test, and the threshold of significance is indicated above (*P < 0.05; **P < 0.01).
