# Supplementary material for: Arabidopsis IQM4, a Novel Calmodulin-Binding Protein, Is Involved With Seed Dormancy and Germination in Arabidopsis
Source: Front Plant Sci. 2018 Jun 5;9:721. doi: 10.3389/fpls.2018.00721 (PMC6008652; doi:10.3389/fpls.2018.00721)
Supplement: Supplementary file 2 [file Table_1.docx]

| **Type** | **Name** | **Sequence (5'→3')** |
| --- | --- | --- |
| *iqm4-1*/*4-2* | IQM4-F1 | ATGGGTCTTTCTCTTTCTTTG |
|  | IQM4-R1 | CTAACAGTTTACTTGAACTC |
|  | LBb1 | GCGTGGACCGCTTGCAACT |
| qRT-PCR | IQM4-F2 | GATTTCGCCTGGTTCTACACG |
|  | IQM4-R2 | GATGAGAAGAGATTGATGAAGC |
| RT-PCR | ATPase-F | CTGAATCAATCTCTTAAGCTGCTG |
|  | ATPase-R | GTGCAGAAAGTTCTACAGAACTAC |
| Y2H | IQM4-F3 | CATATGGGTCTTTCTCTTTCTTTGCTCT |
|  | IQM4-R3 | CCCGGGCTAACAGTTTACTTGAACTCT |
|  | CaM5-F | GAATTCATGGCAGATCAGCTCACCGA |
|  | CaM5-R | GTCGACTCACTTTGCCATCATAACTTT |
| CaM Overlay | IQM4-F4 | CCATGGGCATGGGTCTTTCTCTTTCTTT |
|  | IQM4-R4 | AAGCTTCCGCGCAATCCGCTAAGTTCC |
| BiFC | IQM4-F5 | AAGCTTATGGGTCTTTCTCTTTCTTTGCT |
|  | IQM4-R5 | CCCGGGCTAACAGTTTACTTGAACTCTA |
| Protein Localization | pIQM4-F | GGATCCGACTAATCTTCGTTCGCTCATAT |
|  | pIQM4-R | CCCGGGCTTTCTCTGATTAAACAACAATA |
|  | IQM4-F6 | CCCGGGATGGGTCTTTCTCTTTCTTTGCTC |
|  | IQM4-R6 | CCATGGATCCACAGTTTACTTGAACTCTA |
| IQM4 overexpression | IQM4-F7 | TCTAGAATGGGTCTTTCTCTTTCTTTGCTC |
|  | IQM4-R7 | CCCGGGCTAACAGTTTACTTGAACTCTAG |
| Site-directed mutation | del143-144 | GATGCAGCTGCAACTACGAAGGTGTACAAGAGTTAC |
|  | del143-144antisense | GTAACTCTTGTACACCTTCGTAGTTGCAGCTGCATC |
|  | L143N | GCTTGATGCAGCTGCAACTACGAATCAAAAGGTGTACAAGAGTTACA |
|  | L143Nantisense | TGTAACTCTTGTACACCTTTTGATTCGTAGTTGCAGCTGCATCAAGC |

Table S1 Gene-specific primer sequences and names used in this work
