# Supplementary material for: Arabidopsis IQM4, a Novel Calmodulin-Binding Protein, Is Involved With Seed Dormancy and Germination in Arabidopsis
Source: Front Plant Sci. 2018 Jun 5;9:721. doi: 10.3389/fpls.2018.00721 (PMC6008652; doi:10.3389/fpls.2018.00721)
Supplement: Supplementary file 3 [file Table_2.docx]

Table S2 Gene names and primer sequences used in Real time RT-PCR

| **G**ene | **ID** | **Forward primer (5'→3')** |  | **Reverse primer (5'→3')** |
| --- | --- | --- | --- | --- |
| *ACTIN2* | AT3G18780 | TGACTACGAGCAGGAGATGGAA |  | CAAACGAGGGCTGGAACAA |
| *IQM4* | AT2G26190 | GCCCTAGAGTTCAAGTAAACTG |  | GATGAGAAGAGATTGATGAAGC |
| *FUS3* | AT3G26790 | TTTGTGAATGCTCATGGTCTG |  | TTGTTGTGATGATTAAAATCTTGGA |
| *ABI3* | AT3G24650 | TCCATTAGACAGCAGTCAAGGTTT |  | GGTGTCAAAGAACTCGTTGCTATC |
| *ABI4* | AT2G40220 | TTCTGACATCGAGCTCACTGAT |  | CCCTAACGCCACCTCATGAT |
| *ABI5* | AT2G36270 | AGTCTGCTGCTAGATCTAGAG |  | TGTTGCTTCCTCTTCCTCTCC |
| *LEC1* | AT1G21970 | CACTATCAGCTACCAAAATCCATC |  | TTTTACGCATGATTCTTATGACG |
| *LEC2* | AT1G28300 | TGGCAAGAGAGAGGTGGTTTTC |  | TCCTGTTGATCCTTGCCATCT |
| *WRKY40* | AT1G80840 | GAAGATCCACCGACAAGTGC |  | GCAGTTGCTTCCTCAAGACG |
| *NCED3* | AT3G14440 | AGGTCGCAAGATTCGGGATT |  | GCGGATTTCAGACAGGACACTC |
| *NCED6* | AT3G24220 | CGTTATTCCTATGGAGCAGAATCG |  | GGAGCGAAGTTACCTGATAATTGAA |
| *NCED9* | AT1G78390 | GGAAAACGCCATGATCTCACA |  | AGGATCCGCCGTTTTAGGAT |
| *CYP707A1* | AT4G19230 | TCATCTCACCACCAAGTA |  | AAGGCAATTCTGTCATTCTA |
| *CYP707A2* | AT2G29090 | ATCCATCACTCCTCCGAATTCTTCC |  | TCCATTTCCGAATGGCATGTACG |
| *CYP707A3* | AT5G45340 | CTCTCCGCGGCGGCTCTGTT |  | TCGTGGAGGAGCTACGGCGG |
| *ABI1* | AT4G26080 | AGAGTGTGCCTTTGTATGGTTTTA |  | CATCCTCTCTCTACAATAGTTCGCT |
| *ABI2* | AT5G57050 | GATGGAAGATTCTGTCTCAACGATT |  | GTTTCTCCTTCACTATCTCCTCCG |
| *HAB1* | AT1G72770 | TGCGGTGATTCGAGGGCG |  | TTCCGGTTCTGGGATCACAT |
| *HAB2* | AT1G17550 | CGAGAGATTTCACCTGCAGT |  | CTACATGTCAGCTGCATCAG |
| *SnRK2.2* | AT3G50500 | ATATGCCATCGGGATCTGAA |  | TTGGTTGGGAATGAAGAACAG |
| *SnRK2.3* | AT5G66880 | GTTGGATGGAAGTCCTGCTC |  | TGCCATCATATTCCTGACGA |
| *RAB18* | AT5G66400 | CAGCAGCAGTATGACGAGTA |  | CAGTTCCAAAGCCTTCAGTC |
|  |  |  |  |  |
